# Challenges of COVID-19 prevention during protracted conflicts: differential adherence to preventive measures in “contact line” regions in eastern Ukraine

**DOI:** 10.3389/fpubh.2023.1151452

**Published:** 2023-05-05

**Authors:** Jiho Cha, Jin-Won Noh, Courtland Robinson, Young Dae Kwon, Jinseok Kim

**Affiliations:** ^1^Moonsoul Graduate School of Future Strategy, Korea Advanced Institute of Science and Technology, Daejeon, Republic of Korea; ^2^Division of Health Administration, College of Software and Digital Healthcare Convergence, Yonsei University, Wonju, Republic of Korea; ^3^Department of International Health, Johns Hopkins University Bloomberg School of Public Health, Baltimore, MD, United States; ^4^Department of Humanities and Social Medicine, College of Medicine, The Catholic University of Korea, Seoul, Republic of Korea; ^5^Cathoilc Institute for Public Health and Healthcare Management, The Catholic University of Korea, Seoul, Republic of Korea; ^6^Department of Social Welfare, Seoul Women’s University, Seoul, Republic of Korea

**Keywords:** COVID-19 pandemic, conflict region, preventive measures, conflict-affected populations, Ukraine

## Abstract

**Objectives:**

Despite the epidemiological importance of social vulnerabilities in compliance with preventive measures, little is known about the disproportional nature of preventive behaviors in crisis-affected populations. We examined adherence to COVID-19 preventive behaviors, focusing on social distancing measures in the conflict-affected regions in eastern Ukraine.

**Methods:**

From a multisectoral needs assessment conducted in 2020 using a household interview of a stratified simple random sample, we included 1,617 rural and urban households located in the government-controlled area. We performed multivariable binary logistic regression analysis with latent class analysis (LCA) to identify unmeasured patterns of classification of preventive measures using data from a cross-sectional survey.

**Results:**

The conflict-affected populations showed difficulty in complying with COVID-19 preventive measures due to losses of housing, partners, and access to food resources due to conflicts. Among the various preventive measures, wearing a face mask (88.1%) and washing hands more regularly (71.4%) were the most frequently reported. Compliance with social distancing was significantly lower in those who experienced the direct impacts of conflicts indicated by damaged accommodation or being widowed. Three different groups who showed distinctive patterns of employing COVID-19 preventive measures were identified *via* the LCA model, which were “highly complying group”, “moderately complying group”, and “face masks only group”. The group membership was associated with a respondent’s poverty status.

**Conclusion:**

The findings show the difficulty in compliance with COVID-19 preventive measures among conflict-affected populations indicating secondary impacts of the conflicts on preventive health behaviors. To mitigate the health impacts of conflicts, immediate attention is needed to address barriers to COVID-19 preventive measures among conflict-affected populations in Ukraine. This study suggests the need for public health strategies to improve preventive health behaviors in conflict-affected populations under pandemics or large-scale outbreaks.

## Introduction

The conflict between Russia and Russian-backed separatists and Ukraine began in February 2014. Russia annexed Crimea, and pro-Russian separatists in eastern Ukraine declared independence, leading to eight years of protracted conflict in Donetsk and Luhansk Oblasts (1). In February 2022 when Russia launched a large-scale invasion of Ukraine, humanitarian concerns increased for 18 million people in and near conflict zones in Ukraine, including four million Ukrainian refugees in neighboring countries (1, 2). As the invasion of Ukraine by Russia progressed, civilian casualties included 4,731 killed and 5,900 injured between 24 February to 26 June 2022, in addition to the pre-existing burdens of the COVID-19 pandemic (3).

To understand the dual vulnerabilities of people in these regions, it is necessary to consider that there have already been eight years of protracted conflict, with ceasefire violations along the 428 kilometers of the “contact line” in Donetsk and Luhansk Oblasts (4). Although the magnitude of these violations is not comparable with the contemporary invasion, the civilian populations living by the “contact line” have been exposed to continuous loss of lives and major damage to the social infrastructure. Civilian populations in government-controlled areas (GCA) are physically separated from large urban centers in non-government-controlled areas (NGCA), distressing household economies through marginalization from the labor market and essential social services (5).

Upon detection of its first case of COVID-19, Ukraine swiftly implemented strict containment measures in the spring of 2020. These efforts seemed to prevent a quick and explosive rise in cases throughout the early phase of the pandemic, as seen in other countries (6, 7). Beginning in March 2020, daily case numbers gradually increased until the middle-end of April, hovering between approximately 375–475 daily confirmed cases nationwide through May (8). A similar trend occurred with daily deaths, though these numbers did continue to increase to a peak of 18 deaths per day in mid-May before slowly tapering down (8). Positivity rates during this period were between 3%–10% (9), although there was also poor availability of polymerase chain reaction testing at only 600 tests per day (7). However, along with low compliance with preventive measures in conflict-affected populations, the number of confirmed cases began to increase rapidly as Ukraine eased these restrictions (10). As of April 2, 2022, there have been five million confirmed cases of COVID-19 resulting in 112,000 deaths, which is considerably higher than direct civilian casualties due to the Russian invasion (8).

In response to the devastating effects of the COVID-19 pandemic, furthermore, “closure and containment” policy measures have been implemented in nearly all regions of the world. On top of humanitarian needs from the prolonged conflict, economic distress and unemployment rates were aggravated under the COVID-19 lockdown in Ukraine. The level of disparities in vulnerabilities between those living in GCA and NGCA measurably increased (6, 7).

Despite the epidemiological importance of aggravating social vulnerabilities in health behaviors, however, little is known about the disproportional nature of adherence to COVID-19 preventive behaviors in the context of humanitarian crises. In this study we examined patterns of adherence to COVID-19 preventive measures among conflict-affected populations in GCAs in Ukraine, focusing on identifying differences in adherence patterns among vulnerable groups in the region. Little is known about the disproportional nature of adherence to preventive measures in conflict-affected populations under protracted adverse situations (11, 12).

## Methods

### Data sources and sampling methods

This study is based on a secondary analysis of the public dataset from the original mixed methods study of a cross-sectional survey research design, Multisectoral Needs Assessment (MSNA), which was carried within the framework of the Inter-Cluster Coordination Team of the United Nations Office for the Coordination of Humanitarian Affairs (UN-OCHA) and co-facilitated by Renewed Efforts Against Child Hunger and undernutrition (REACH) Initiative, in close collaboration with the UN-OCHA. To provide humanitarian planning among international and national actors in Ukraine, the MSNA was designed to investigate the multi-sector needs of the conflict-affected populations in eastern Ukraine in 2020. Further to prolonged conflicts restricting the essential movement of people and goods in Donetsk and Luhansk Oblasts, COVID-19 and its containment measures have led to an economic downturn damaging the livelihood of civilians along the contact line in eastern Ukraine. The vulnerability of conflict-affected populations, especially those over the age of 60 years, was further compounded by higher health risks and inadequate access to livelihoods and essential items. Despite the humanitarian concerns, the international travel restriction also limited the accessibility of international actors in this settlement area. There was the potential risk of the field survey increasing the likelihood of COVID-19 transmission between interviewers and respondents.

Reflecting this population of interests, this study targeted the civilian population in GCA with the specific inclusion criteria of the displaced and non-displaced persons who are residents in settlements smaller than 100,000 people within 20 km of the contact line. Between July 29 and August 15, 2020, structured face-to-face interviews were conducted with heads of households using stratified simple random sampling. Any individuals under the age of 18 were excluded from the interview.

A statistically representative sample of 1,617 rural and urban households in GCAs of Donetsk and Luhansk Oblasts was collected according to distance to the contact line (0–5 km and 5–20 km) with a 95% confidence level. To create a representative sample of the population of interest (95% confidence level, 5% margin of error for each stratum), a total of 1,617 households were selected following strata: 404 were sampled from 0 to 5 km urban areas representing 211,857 persons in 22 settlements and 402 rural households representing 39,003 in 65 settlements. In regions 5–20 km from the contact line, 404 urban households were sampled including 230,712 persons in 37 settlements and 407 rural households including 89,408 persons in 207 settlements.

Based on the population data of the State Statistics Service of Ukraine, the weight of a random point selection in each region was calculated using QGIS to reflect a higher-density area. During the field data collection, the nearest household in a computerized random point was identified and visited. Household head or another key household member were interviewed on behalf of the household.

A face-to-face interview was conducted by interviewers in REACH Initiative. Prior to data collection, interview techniques were trained with an emphasis on the protection issues of vulnerable populations during the interviews. Following field interviews, there were workshops to gather the direct observations of interviewers on humanitarian environments and challenges of the respondents in the settlement. Given the epidemiological situation of COVID-19, the face-to-face interview was designed and implemented with COVID-19 mitigation measures to reduce the risk to staff and respondents. All interviewers and relevant field staff were trained to adhere to COVID-19 prevention protocols to minimize the transmission risk between survey respondents. Regarding the study protocol, the Health Cluster Ukraine concluded face-to-face surveys could be conducted in the regions.

### Variables and measurements

Key variables in the MSNA were previously defined in coordination with UN-OCHA’s cluster coordinators and cover types and magnitude of humanitarian needs in sectors of protection, shelter and non-food items, health, food security and livelihoods, water, sanitation and hygiene, and education. To identify different patterns of COVID-19 preventive measures and their associations with vulnerabilities in conflict-affected populations, we focused on socio-demographic and humanitarian factors which were associated with adherence to COVID-19 preventive measures.

In our analysis, key outcome variables of interest were “COVID-19 preventive behaviors” including “reducing movement outside the house”, “stopping physical contact”, “keeping distance from people”, “avoiding public places and gatherings”, “avoiding public transport”, “wearing a face mask”, and “washing hands”. It was ascertained by the question, “Since you heard about COVID-19, have you and your household members taken any action to prevent yourselves from getting COVID-19?,” with possible responses chosen all that applicable. Twelve individual items of “COVID-19 preventive behaviors” were developed to evaluate a series of preventive policy measures across countries and comparability was achieved by measuring with the same questions in countries such as Afghanistan, Ukraine, Bangladesh, the Central African Republic, Nigeria, Iraq, and Libya. To ensure national representativeness, all responses were reviewed and validated by IMPACT Initiatives according to data protection standard operating procedures and the data cleaning guidelines (13). To test potential associations with patterns of COVID-19 preventive measures, items related to economic status household income, employment status, and debt status (i.e., “Is the head of household currently in debt?”) were used as key independent variables along with other socio-demographic factors such as gender, age, and marital status. Similarly, to examine its association with preventive behaviors, we analyzed items related to food security (i.e., “In the past 30 days, was there ever no food to eat of any kind in your house because of lack of resources to get food?”) which was adapted from the widely accepted household hunger scale in UN, United States Agency for International Development, and other international actors. In addition, we considered humanitarian factors related to displacement status (i.e., “Is the head of household displaced as a result of conflict?”) and accommodation damage status (i.e., “Does the shelter currently have any conflict-related damage or defects?”) as indicators of more direct impacts of conflicts and analyzed as key independent variables for the preventive behaviors.

### Statistical analysis

To produce unbiased estimates of population characteristics, Taylor-series linearization was employed for variance estimation to account for the complex sample design of multi-sector needs assessment (MSNA). Strata with a single sampling unit are centered at the overall mean instead of the strata mean in this procedure. Descriptive analysis was conducted to summarize each household’s socio-demographic characteristics, humanitarian characteristics, and other key variables of interest. Results are presented as sample frequencies with weighted percentage or weighted mean estimates with 95% confidence intervals (CIs) as appropriate. Inter-group comparisons were performed using the Rao-Scott corrected chi-square test for categorical data and sampling design weighted univariable linear regression for continuous data.

For dichotomous outcomes, multivariable binary logistic regression analysis was performed. The adjusted coefficients with 95% CI estimates were reported. Latent class analysis (LCA) was also conducted to identify unmeasured patterns of classification of COVID-19 preventive measures using categorical observed variables of COVID-19 in MSNA 2020. The data were analyzed using Stata/MP version 16.1 (StataCorp LP, College Station, TX, United States). The alpha level of 0.05 (two-tailed) was considered the threshold for statistical significance.

### Ethical consideration

This study was conducted with ethics approval by the Institutional Review Board of Dankook University (IRB No. DKU-IRB-NON2020-006) in the category of human-subject database research. To adhere to the principle of doing no harm, potentially sensitive questions were not included in the survey. The survey was not conducted with individuals under the age of 18. Prior to conducting the survey with the target population, interviewers were trained to ensure the protection of vulnerable populations along with relevant training of interviewing techniques in the humanitarian context. Interviewers provided information about the purpose of the survey and obtained consent from participants. The study was designed to minimize the collection and storage of personally identifiable information, limit access to personal data, and assign formalized and limited access rights to individuals who have access to datasets containing personal information.

## Results

The summary of the sample characteristics is presented in Table 1. Most of the heads of households were females (72.0%). The average age of the household heads was 56.7 years (SD = 15.3). About half (47.8%) were married or accompanied, 30.6% widowed, 13.6% divorced or separated, and 8.0% single. Almost 30% of the household heads were working for pay, 9.2% were not working, 8.4% were doing household chores, and 53.6% were retired or disabled. The average and median monthly household income was 3444.0 (SD = 3017.2) and 2,500 Ukrainian hryvnia (UAH), respectively. Over one out of five (22.0%) answered that they had debt of which average and median were 1271.3 (SD = 5065.2) and 0 UAH, respectively. Fewer than one out of ten respondents (8.4%) reported that they had experienced displacements from home because of the conflict, and over a quarter of households (26.8%) had experienced conflict related damage to their shelter (Table 1).

**Table 1:** Sample characteristics (*n* = 1,617).

Characteristics	Frequency	Percentage (%)
Sex		
Female	1,164	72.0
Marital status		
Married/companion	773	47.8
Divorced/separate	220	13.6
Widowed	495	30.6
Single	129	8.0
Displacement		
Yes	137	8.5
Employment status		
Housework	136	8.4
Paid work	465	28.8
Not working	149	9.2
Retired/disabled	867	53.6
Debt		
Yes	353	22.0
Damaged accommodation		
Yes	434	26.8
Under the poverty level (<$3.2 per day)	787	50.9
**Variable**	**Mean**	**SD**
Age (year)	56.7	15.3
Income (UAH)	3444.0	3017.2
Current debt (UAH)	1271.3	5065.2

SD, standard deviation; UAH, Ukrainian hryvnia.

Table 2 summarizes the distributions of various preventive measures that people employed to keep them safe from COVID-19. Wearing a face mask (88.1%) and washing hands more regularly (71.4%) were the most common measures, followed by keeping distance from people (40.8%), reducing movement outside the house (34.8%), keeping surfaces clean (31.9%), avoiding public places and gatherings (29.5%), avoiding public transport (16.6%), stopping handshakes or physical contact (16.6%), and praying to god (10.3%). Less than 10% were wearing gloves (9.2%), not leaving the house at all (3.3%), or staying away from animals (1.3%) (Table 2).

**Table 2:** Distribution of COVID-19 preventive measures employed by survey participants (*n* = 1,617).

Variable	Category	Frequency	Percentage (%)
Actions to prevent COVID-19	Wearing a face mask	1,425	88.1
	Washing hands more regularly	1,154	71.4
	Keeping distance from people	660	40.8
	Reducing movement outside the house	562	34.8
	Keeping surfaces clean	516	31.9
	Avoiding public places and gatherings	477	29.5
	Avoiding public transport	269	16.6
	Stopping handshakes or physical contact	268	16.6
	Praying to God	167	10.3
	Wearing gloves	149	9.2
	Not leaving the house at all	53	3.3
	Staying away from animals	21	1.3

Our analysis revealed that there were significant differences in terms of the adoption of preventive measures between displaced and nondisplaced persons, including compliance with social distancing and other policies related to limits on human mobility (stopping or reducing movements outside the house, avoiding public transport, etc.). In addition, social distancing measures were less often adopted by those who experienced direct impacts of conflicts such as damaged accommodation. Widows also showed less compliance with social distancing measures. Not surprisingly, access to food resources was negatively associated with adherence of social distancing measures (Table 3).

**Table 3:** Results of the logistic regression of employment of social distancing measures (*n* = 1,537).

Characteristics	Coefficient (B)	S.E. (B)	95% CI
Sex	0.38^**^	0.14	(0.11, 0.65)
Female			
Age	<0.01	0.01	(−0.01, 0.01)
Income	<0.01	0.00	(0.00, 0.00)
Debt			
Yes	0.19	0.14	(−0.09, 0.46)
Marital status			
Divorced/separate	−0.03	0.18	(−0.38, 0.31)
Widowed	−0.33^*^	0.15	(−0.63, −0.03)
Single	−0.51^*^	0.22	(−0.93, −0.08)
Displacement	0.28	0.21	(−0.14, 0.69)
Employment status			
Housework	−0.08	0.27	(−0.61, 0.46)
Paid work	0.09	0.23	(−0.36, 0.54)
Retired/disabled	−0.03	0.23	(−0.49, 0.42)
No food resources	−0.39^*^	0.19	(−0.77, −0.01)
Damaged accommodation	−0.55^***^	0.13	(−0.81, −0.29)
Chi^2^ (13)	45.05^***^		
Pseudo *R*^2^	0.02		
Model log-likelihood	−949.92		

^*^p < 0.05; ^**^p < 0.01; ^***^p < 0.001; S.E., standard error; CI, confidence interval.

COVID-19 preventive measures were subjected to the LCA model. Out of 12 preventive measures, “staying away from animals” was excluded due to extremely low prevalence (1.3%). The Lo–Mendell–Rubin likelihood ratio test (LMR-LRT) of the four-class model (df = 1999; LR chi-squared = 843.43; entropy = 0.695; Bayesian information criterion = 15416.06; LMR-LRT = 206.31; *p* = 0.115) was insignificant, which indicated that the three-class model (df = 2010; LR chi-squared = 1107.20; entropy = 0.773; Bayesian information criterion = 15536.29; LMR-LRT = 365.85; *p* < 0.001) would be adequate.

**Figure 1:**
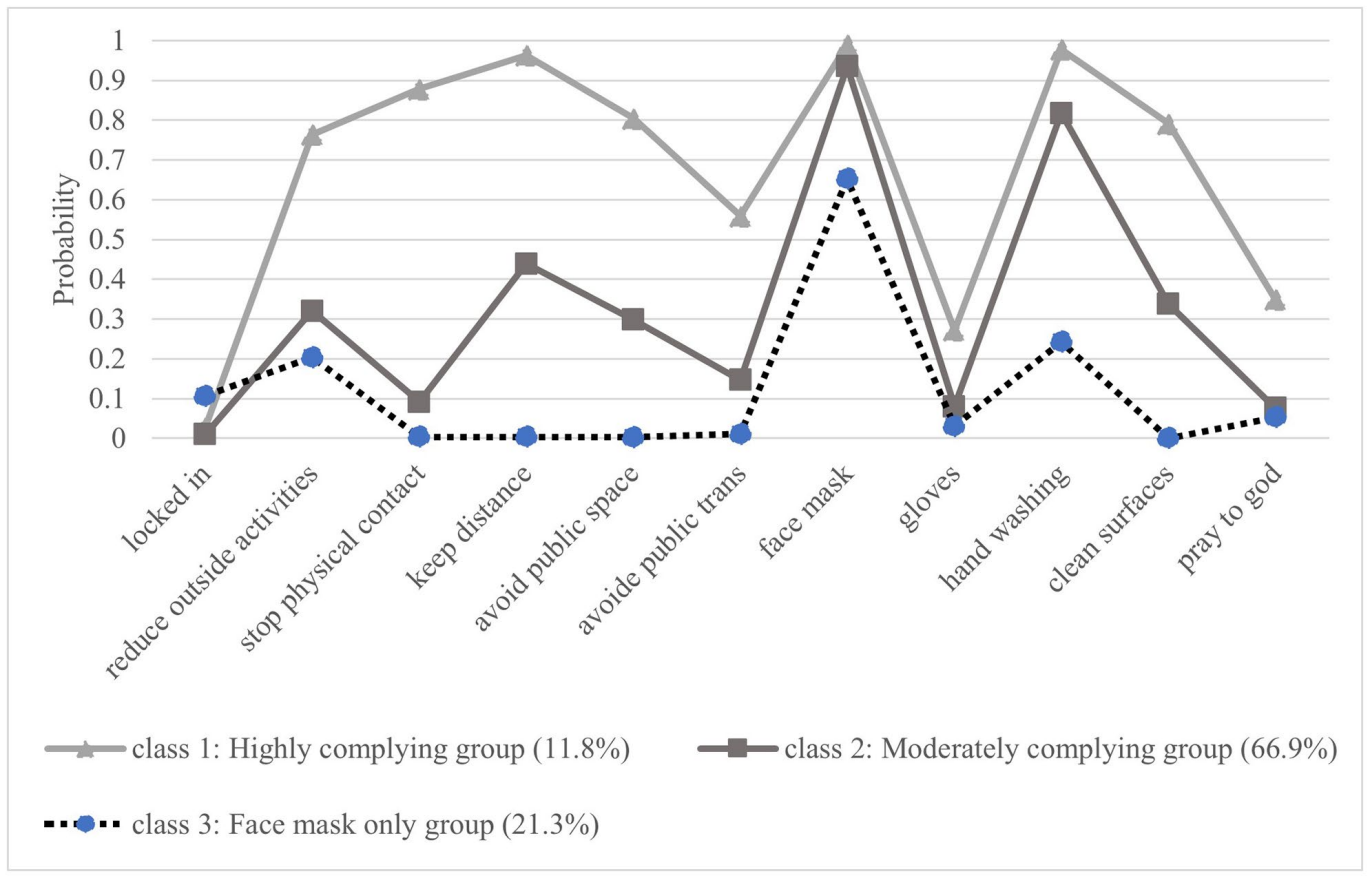
Latent class analysis results for Coronavirus disease-19 preventive measures.

Figure 1 presents a visual representation of the preventive measures obtained from the LCA. The *y*-axis represents the estimated probability that the household heads employed a particular preventive measure (*x*-axis) against COVID-19. In Figure 1, we labeled class 1 as the “highly complying group”, class 2 as the “moderately complying group”, and class 3 as the “face masks only group”. The “highly complying group” employed all preventive measures but “locked-in” more frequently than any other group. The respondents in the “moderately complying group” were less likely than the “highly complying group”, yet moderately likely to utilize social distancing-oriented measures such as “reducing outside activities” or “keeping distance” but utilizes personal hygiene-oriented measures such as “wearing face masks” or “hand washing” at similar levels as the “highly complying group”. The “face masks only group” utilized almost exclusively personal hygiene-oriented measures but were less likely to do so than other groups (Figure 1).

To further understand the nature of the LCA groups, we regressed class membership on the poverty measure meaning earning less than $3.2 per day. Results showed that those who were below the poverty level were less likely to be members of the “moderately complying group” (B [SE] = − 0.64 [0.16], *p* < 0.001) or of “face mask only group” (B [SE] = −0.61 [0.21], *p* = 0.005) than of the “highly complying group” (Table 4).

**Table 4:** Latent class regression analysis results.

Latent class analysis group	Coefficient (B)	S.E. (B)	95% CI
“Highly complying group” (reference)			
“Moderately complying group”			
Under the poverty level	−0.64^**^	0.16	(−0.96, −0.32)
“Face masks only group”			
Under the poverty level	−0.61^*^	0.21	(−1.03, −0.19)

^*^p < 0.01; ^**^p < 0 0.001; S.E., standard error; CI, confidence interval.

## Discussion

This study analyzed the patterns of adherence to COVID-19 preventive measures among conflict-affected populations in GCAs in eastern Ukraine. Ukraine recorded its first case of COVID-19 on February 29, 2020, in Chernivtsi Oblast, eventually experiencing five million confirmed cases and 112,000 deaths as of April 2, 2022 (7). To curb the spread of the disease, the country implemented stringent policy measures until the Russian invasion, including border closures, international and national travel restrictions, forced cancellation of public gatherings, gathering size restrictions, nationwide school closures, and finally workplace closures (6, 7). Concurrently, the government issued a recommended stay-at-home order (9). Most notably, the Ukrainian government fully shut down movement across the contact line, making it impossible for those living in GCAs to move to NGCAs and preventing approximately 900,000 people from crossing the contact line every month (7).

“Closure and containment” policies aggravated the vulnerability of civilian populations in GCAs by isolating them from the rest of Ukraine (14). Such policies led to a range of negative secondary impacts, including widespread economic insecurity and poor access to healthcare (14, 15). The effective shutdown of the country put enormous social and economic burdens on its residents, including high rates of job loss and decreased incomes, increased food insecurity, and decreased access to healthcare, among others (14).

Among conflict-affected populations in Ukraine, our results showed significant differences in adherence to COVID-19 preventive measures, especially for social distancing and other policies restricting human mobility. The level of disparities was associated with pre-existing vulnerabilities of conflict-affected populations in the GCAs. Compliance with social distancing was significantly lower in those who experienced the direct impacts of conflicts, such as those in damaged accommodations and widows. Food security was also significantly associated with compliance with social distancing measures. As our analysis showed, social distancing measures were more affected than other preventive measures such as wearing masks or hand washing. The “highly complying group” had less chances to earn household income over the poverty level ($3.2 per day). Unsurprisingly, socioeconomic burdens seem to have been disproportionately high for those affected by dual burdens of COVID-19 and conflicts. These findings are consistent with those of a prior study indicating that adherence to social distancing in Congo was positively associated with each province’s socioeconomic status, being higher in provinces with greater human, logistical, and financial resources than other provinces (16). Countries around the world are struggling to balance positive public health achievements with the negative economic, social costs from preventive measures against COVID-19 (16). In particular, low resource countries that may have to wait until 2023 for widespread immunization have to support appropriate stop-gap measures for vulnerable populations (17, 18).

We fully recognize the limitations of this study, which was conducted using quantitative data collected in 2020, during the early phase of the COVID-19 pandemic. This study does not include any data related to the direct impacts of the contemporary invasion by Russia on the preventive behaviors for the COVID-19 pandemic or other infectious diseases. Therefore, our analysis is less generalizable on those disease-preventive behaviors under the escalation of the war since the Russian invasion of Ukraine in February 2022. In terms of the measurement, the 12-item measure of “COVID-19 preventive behaviors” was limited because it was not a validated instrument. However, to the best of our knowledge, there is an absence of an appropriate instrument that can comprehensively capture a series of preventive policy measures in such a specific situation. Therefore, the 12-item “COVID-19 preventive behaviors” questionnaire was constructed although it was not considered a validated instrument. We did not perform a psychometric test to ensure the reliability and validity of the items but analyzed the items individually.

Furthermore, the original study was not designed to evaluate mortality in conflict-affected populations. Although we provide evidence of compliance patterns for preventive measures, it is still unknown about the complex impacts of prolonged conflicts on COVID-19-related deaths. In Ukraine, the COVID-19 pandemic has caused more than 112,000 deaths as of April 2, 2022 (8). Notably, the reported number of COVID-19 deaths is far greater than civilian casualties due to the invasion by Russia, which has caused 4,731 civilian deaths and 5,900 injuries from 24 February to 26 June 2022 (3). A further quantitative dataset is needed to measure the direct or indirect impacts of the conflicts on mortality related to COVID-19 or other infectious diseases.

Lastly, we also fully understand impacts of the prolonged conflicts on health are complex and heterogeneous, and these are hard to be measured with quantitative data alone. This study defined the conflict-affected populations of interest as “displaced and non-displaced households residing in settlements smaller than 100,000 people and located within 20 km of the contact line,” considering the complexity of both direct (physical injuries on body, mind, and house, displacement) and indirect (socioeconomic impacts such as unemployment, etc.) impacts of war. Also, specifically, we tried identifying households with more direct loss or injuries from the conflicts, using items related to displacement status and accommodation damage status by the conflict as indicators. Nonetheless, it is out of scope for this quantitative analysis to examine the perspectives and lived experiences of the conflict-affected groups about the complex impacts of the conflicts on their everyday challenges and specifically on preventive behaviors on COVID-19. Further qualitative analysis is needed to understand complex and heterogeneous pathways of lower access to preventive measures in conflict-affected populations.

## Conclusion

This study provides evidence about compliance with the preventive behaviors during the COVID-19 pandemic in the protracted conflict areas of Donetsk and Luhansk Oblasts, where the chronic loss of life and damage to the social infrastructure were aggravated by the COVID-19 pandemic. Our study results imply that the conflict-affected populations find it challenging to comply with COVID-19 preventive measures due to losses of housing, partners, or access to food resources under direct or indirect impacts of conflicts. Social distancing can aggravate livelihoods as household incomes often decrease. Along with low vaccination rates in conflict-affected regions, low compliance with social distancing and other preventive measures could result in higher prevalence and mortality of COVID-19 forming a dual burden alongside civilian casualties due to the conflicts between Russian and Ukraine (19). Further attention on those complex health impacts is required to evaluate civilian causalities of the Russian invasion of Ukraine.

The dual impacts of the conflict and pandemic on human health are rarely measurable mainly due to the lack of available dataset in humanitarian contexts. The changing preventive health behaviors in the conflict-affected population is still not considered in the modifiable target of pandemic and other global health responses. This research provides meaningful results on secondary socioeconomic impacts of the conflicts on preventive health behaviors and suggests public health needs to provide immediate attention and coping strategies for improving human health behaviors in conflict-affected populations. Further research is required for evaluating complex political, socioeconomic, and cultural impact pathways of conflicts on preventive behaviors, especially in the contexts of pandemics or other large-scale outbreaks in conflict-affected populations.

## Data availability statement

Publicly available datasets were analyzed in this study. This data can be found at: https://www.reachresourcecentre.info/search/?search=1&initiative%5B%5D=reach&pcountry%5B%5D=ukraine&ptheme%5B%5D=multi-sector-assessments&dates=&keywords=.

## Ethics statement

The studies involving human participants were reviewed and approved by Dankook University (IRB No. DKU-IRB-NON2020-006). Written informed consent for participation was not required for this study in accordance with the national legislation and the institutional requirements.

## Author contributions

JC, J-WN, JK, and YK take responsibility of study design. JK conducted data extraction and data analysis. JC, J-WN, JK, CR, and YK wrote the first draft of the manuscript. J-WN and YK revised the manuscript for important intellectual content. YK and JK supervised all the activities. All authors contributed to the article and approved the submitted version.

## Conflict of interest

The authors declare that the research was conducted in the absence of any commercial or financial relationships that could be construed as a potential conflict of interest.

## Publisher’s note

All claims expressed in this article are solely those of the authors and do not necessarily represent those of their affiliated organizations, or those of the publisher, the editors and the reviewers. Any product that may be evaluated in this article, or claim that may be made by its manufacturer, is not guaranteed or endorsed by the publisher.
